# Acceptability of Long-Acting Injectable Antiretroviral Therapy Among People with HIV Receiving Care at Three Ryan White Funded Clinics in the United States

**DOI:** 10.1007/s10461-024-04315-0

**Published:** 2024-04-10

**Authors:** Xavier A. Erguera, Kimberly A. Koester, Manami Diaz Tsuzuki, Kaylin V. Dance, Rey Flores, Jared Kerman, Moira C. McNulty, Jonathan A. Colasanti, Lauren F. Collins, Elizabeth T. Montgomery, Mallory O. Johnson, John A. Sauceda, Katerina A. Christopoulos

**Affiliations:** 1 Division of HIV, ID and Global Medicine, Department of Medicine, University of California San Francisco, San Francisco, CA USA; 2 Department of Public Health, Zuckerberg San Francisco General Hospital, San Francisco, CA USA; 3 Division of Prevention Science, Department of Medicine, Center for AIDS Prevention Studies, University of California San Francisco, San Francisco, CA USA; 4 School of Medicine, University of California San Francisco, San Francisco, CA USA; 5 Division of Infectious Diseases, Department of Medicine, Emory University School of Medicine, Atlanta, GA USA; 6 Ponce de Leon Center, Grady Health System, Atlanta, GA USA; 7 Department of Family and Community Medicine, College of Medicine, University of Illinois Chicago, Chicago, IL USA; 8 Cancer Treatment Centers of America, Comprehensive Care and Research Center, City of Hope Chicago, Chicago, IL USA; 9 Chicago Center for HIV Elimination, University of Chicago, Chicago, IL USA; 10 Section of Infectious Diseases and Global Health, Department of Medicine, University of Chicago, Chicago, IL USA; 11 Women’s Global Health Imperative, RTI International, Berkeley, CA USA; 12 Department of Epidemiology and Biostatistics, School of Medicine, University of California San Francisco, San Francisco, CA USA; 13 Division of HIV, Infectious Disease, and Global Medicine, San Francisco General Hospital, 1001 Potrero Avenue, Building 80, Room 424, San Francisco, CA 94110 USA

**Keywords:** Long-acting injectable antiretroviral therapy, LAI ART, Cabotegravir-rilpivirine, Acceptability, Qualitative research, Implementation science

## Abstract

Understanding the acceptability of long-acting injectable antiretroviral therapy (LAI-ART) among people with HIV (PWH), especially priority populations, is essential for effective implementation. We conducted semi-structured interviews with patients in three Ryan White-funded HIV clinics in San Francisco, Chicago, and Atlanta. We employed maximal variation sampling across age, gender, race, ethnicity, and time living with HIV and oversampled for individuals with suboptimal clinical engagement. An 8-step hybrid deductive and inductive thematic analysis approach guided data analysis. Between August 2020 and July 2021, we conducted 72 interviews. Median age was 46 years; 28% were ciswomen, 7% transwomen, 44% Black/African-American and 35% Latinx, 43% endorsed a psychiatric diagnosis, 35% were experiencing homelessness/unstable housing, and 10% had recent substance use. Approximately 24% were sub-optimally engaged in care. We observed a spectrum of LAI-ART acceptability, ranging from enthusiasm to hesitancy to rejection. We also characterized four emergent orientations towards LAI-ART: innovator, pragmatist, deliberator, and skeptic. Overall, the majority of participants expressed favorable initial reactions towards LAI-ART. Most approached LAI-ART pragmatically, but acceptability was not static, often increasing over the course of the interview. Participants considered their HIV providers as essential for affirming personal relevance. HIV stigma, privacy concerns, and medical mistrust had varied impacts, sometimes facilitating and other times hindering personal relevance. These findings held across priority populations, specifically young adults, cis/trans women, racial/ethnic minorities, and individuals with suboptimal clinical engagement. Further research is needed to explore the transition from hypothetical acceptance to uptake and to confirm the actual benefits and drawbacks of this treatment.

**Supplementary Information:**

The online version contains supplementary material available at 10.1007/s10461-024-04315-0.

## Resumen

La aceptabilidad de la terapia antirretroviral inyectable de acción prolongada (LAI-ART, por su sigla en inglés) entre personas con VIH es esencial para una implementación efectiva. Durante el periodo de agosto de 2020 a julio de 2021, realizamos 72 entrevistas semiestructuradas con personas con VIH en clínicas públicas ubicadas en San Francisco, Chicago y Atlanta. Un análisis temático, tanto deductivo como inductivo, guio nuestra investigación. Observamos un espectro de aceptabilidad de LAI-ART que va desde el entusiasmo hasta la indecisión y el rechazo. También caracterizamos cuatro orientaciones actitudinales emergentes hacia LAI-ART: innovadora, pragmática, deliberativa y escéptica. Los participantes también señalaron la importancia de sus proveedores de VIH para validar su relevancia personal. El estigma asociado al VIH, preocupaciones sobre la privacidad y desconfianza en el sistema médico tuvieron diversos impactos, a veces facilitando y otras veces obstaculizando la relevancia personal. Entre las poblaciones prioritarias del estudio, los resultados fueron consistentes.

## Introduction

Effective human immunodeficiency virus (HIV) treatment is essential for preserving the health of people with HIV (PWH) [1]. However, sustained viral suppression, a critical goal for both disease management and prevention, remains challenging [2–8]. Moreover, priority populations that are disproportionately affected by the HIV epidemic, including gender and sexual minorities, racial and ethnic minorities, and individuals facing adverse social determinants of health, often experience suboptimal virologic control from oral antiretroviral therapy (ART) adherence challenges and bear a disproportionate burden of the disease [9–12]. Long-acting injectable antiretroviral therapy (LAI-ART) presents a promising option to reduce these disparities [13, 14]. In January 2021, the US Federal Drug Administration approved the first LAI-ART formulation, long-acting injectable cabotegravir–rilpivirine (CAB/RVP-LA), as a switch regimen for PWH with stable viral suppression on oral ART. However, early data from real-world implementation of CAB/RVP-LA suggest potential efficacy for individuals with adherence challenges and detectable viremia [15].

The pre-implementation phase of a new innovation is an important albeit understudied stage in implementation research. Early insights into motivations for and barriers to uptake of efficacious interventions can help guard against challenges that may stymie implementation success and provide new lenses through which to view implementation efforts [16, 17]. Surveys of PWH prior to the clinical availability of LAI-ART demonstrated substantive hypothetical willingness to use this treatment alternative, ranging from 55 to 88%, with the highest interest observed among adolescents, young adults, and individuals facing adherence challenges [18–22]. The desire to lessen adherence concerns and the convenience of non-daily dosing emerged as primary motivators for considering LAI-ART, underscoring the weight imposed on PWH by daily oral ART regimens [21, 22]. In contrast, familiarity with oral ART and routine daily dosing ability may contribute to lower rates of hypothetical willingness [23], along with concerns about potential side effects [24], feasibility of more frequent clinic visits to accommodate injection administration [25, 26], and medical mistrust of healthcare systems [27].

Qualitative methods are particularly effective for understanding the early response to innovations, weighing of pros and cons, and ascertaining affective attitudes [16, 17]. Interviews with racial/ethnic minority participants in the Women's Interagency Health Study revealed an enthusiastic preference for LAI-ART over pills due to perceived effectiveness, confidentiality, and convenience [28], especially among those with a history of injectable medication use [29]. However, these findings were specific to a longitudinal sample of predominantly older ciswomen aged ≥ 50 years. In another investigation, young adults from diverse regions of the US showed significant interest in LAI-ART, but their enthusiasm was dampened by needle aversion, concerns about past injection drug use, and a notable preference for better tolerated oral ART options, such as smaller pill size and gummy forms [30]. Interviews with PWH experiencing substance use and/or housing instability revealed a preferrence for LAI-ART but voiced concerns about efficacy, safety, and logistical barriers [31], with those virally suppressed on oral ART regimens being reluctant to switch therapies [32].

Our research explored LAI-ART acceptability among a socio-demographically diverse sample of PWH receiving care in clinics for the underserved, including young adults, cis/trans women, racial/ethnic minorities, and individuals with suboptimal clinical engagement. Specifically, we sought to analyze participants’ narratives to examine how PWH come to understand and approach decision-making regarding LAI-ART.

## Methods

### Study Setting and Population

The study was conducted at three Ryan White funded HIV clinics affiliated with academic medical centers: Ward 86 at San Francisco General Hospital, the University of Chicago Infectious Disease Program (IDP), and the Ponce de Leon Center of the Grady Health System in Atlanta, Georgia. Both Ward 86 and the Ponce Center are part of urban safety-net hospital systems, serving low-income or uninsured patients, while approximately two-thirds of patients at IDP have public insurance.

### Research Team

A multidisciplinary team conducted the study, including a medical anthropologist (KAK), health psychologists (MOJ, JAS), a socio-behavioral epidemiologist (ETM), physician-researchers (KAC, MCM, JAC, LFC), and research coordinators experienced in qualitative interviewing (XAE, MDT, KVD, RF, JK). The team reflects diverse gender, racial, and ethnic identities, as well as bilingual English–Spanish language skills.

### Study Participants and Sampling

Eligible participants were aged ≥ 18 years, receiving HIV care at the clinic for the past year, and fluent in English or Spanish. Recruitment occurred within the clinics via multiple methods. Providers were made aware of the study in clinical meetings and by email and told they could refer patients to the study team. Research coordinators were also stationed in or near the clinic to accept on-the-spot referrals from providers and staff. Study flyers were posted in the clinic and in research spaces adjacent to the clinic. Research coordinators followed a standard script to offer study participation, which was described as an opportunity for patients to share thoughts and perspectives on forthcoming injectable HIV treatment options. We used maximal variation sampling to ensure diversity across age, gender, race, ethnicity, and time with HIV. Specific enrollment goals included ≥ 30% cis/trans women to account for the fact that the proportion of women at the three clinics ranged from 15%-40%, 20 Spanish-monolingual patients from Ward 86 to complement the other sites which primarily serve Black/African-American individuals, and an oversampling of individuals with suboptimal clinical engagement (≥ 2 missed HIV care appointments in the past year or < 2 HIV care appointments ≥ 90 days apart in the past year with a detectable viral load ≥ 200 copies/ml) given the potential role of LAI-ART in those with adherence challenges.

### Data Collection

Data collection involved one-time interviews using a semi-structured guide (see Supplementary Information) to explore influential factors across the socio-ecological model of HIV care engagement [33] (i.e., individual, interpersonal, community, and larger societal contexts). The guide covered HIV care history, treatment experience, and LAI-ART perceptions, preferences, and intentions, with attention to different delivery options (i.e., where to receive injections, appointments vs drop-in, and injection schedule) and anticipated prescription requirements (i.e., viral suppression prior to LAI-ART initiation, use of an oral lead-in, and potential for oral ART bridging in the case of late injections). The guide was translated into Spanish, piloted with three individuals (in both English and Spanish), and refined for clarity and flow. To explain LAI-ART, we utilized an educational script (see guide in Supplementary Information) developed by physicians and vetted by patients with prior experience through compassionate use [34]. The script emphasized the long-acting injections replaced daily oral pills, consisted of two injections into the buttock muscles containing two different medications, described common side effects, and reviewed in the event of discontinuation of injections that resumption of daily oral ART would be necessary to prevent drug resistance.

Research coordinators at each site conducted interviews either in-person, utilizing private spaces within the clinic, or, when COVID precautions were active, via videoconferencing. Interviews were audio-recorded, transcribed verbatim and translated from Spanish to English as-needed. They lasted 60–90 min, were followed by a short demographic survey, and participants received a $50 reimbursement. Afterwards, research coordinators drafted fieldnotes to record impressions, non-verbal observations, and provide a synopsis of the interview. They also extracted appointment and laboratory data (CD4 count and HIV viral load) from electronic medical records. Regular debriefing meetings helped foster a shared analytical perspective and directed data collection efforts [35].

### Data Analysis

**Fig. 1:**
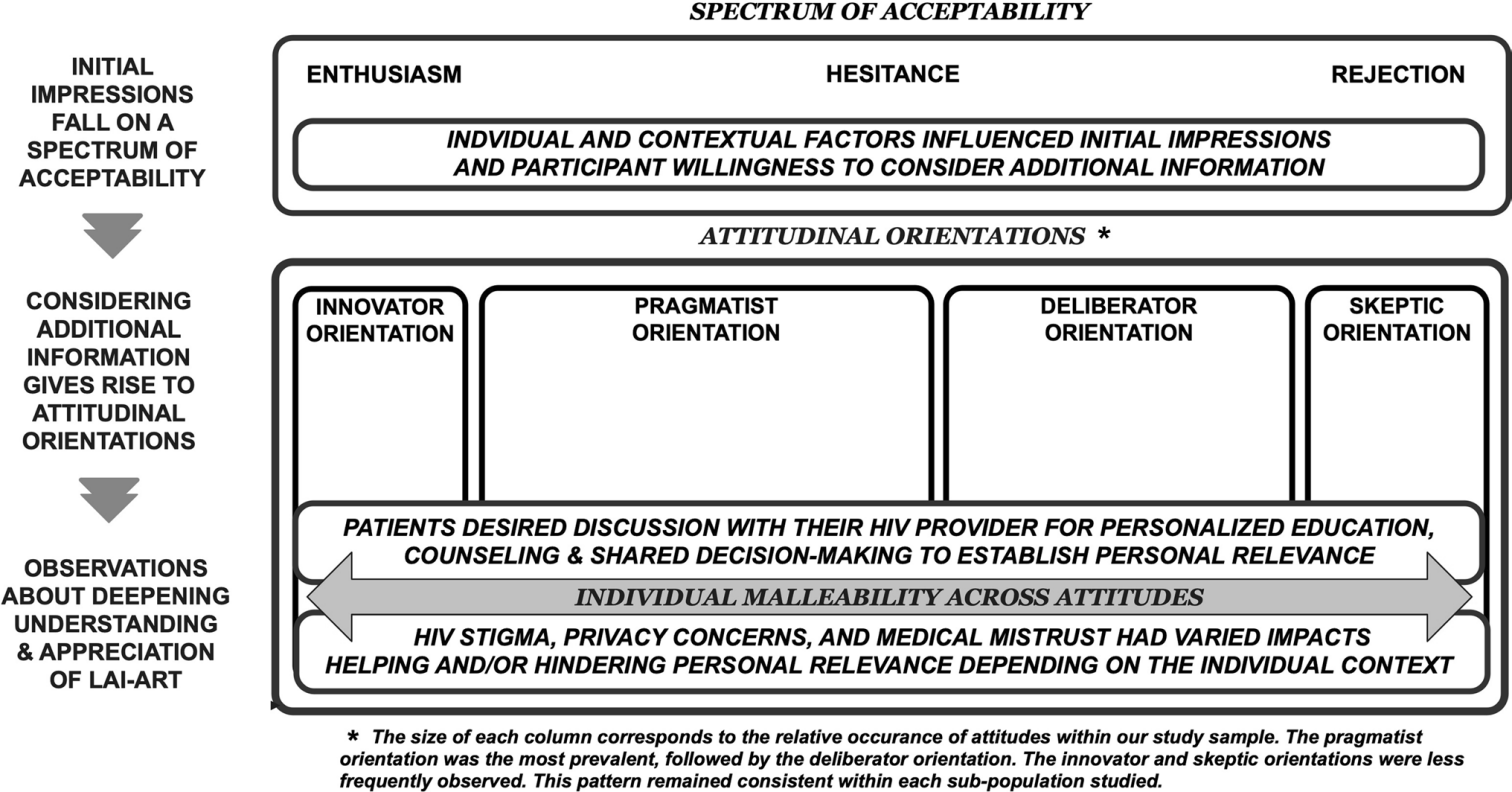
LAI-ART acceptability themes and sub-themes

We expanded Fereday and Muir-Cochrane's hybrid thematic analysis approach [36] into an 8-stage process: familiarization, code development, code application, summarization, identifying initial themes, conceptual refinement, theme corroboration, and contextualization. Rapid-analysis insights and open coding of three interviews generated preliminary codes that were refined using an additional three interviews (XAE, MDT), resulting in 78 codes. Dedoose, a qualitative data analysis platform, (Version 9.0.90, 2023) facilitated coding and analysis [37]. Primary coding was conducted by a single research coordinator (XAE) and secondary coding by other research coordinators (MDT, KVD, RF, JK), resolving disagreements through consensus. In this analysis we focus on seven codes: acceptability, motivation, concerns, uptake intentions, privacy, stigma, and medical mistrust. Essential points were distilled and summarized in a table, which guided the generation of cross-site memos and identification of preliminary themes (XAE, MDT, KVD). To ensure comprehensive coverage of preliminary themes, a re-reading of 15 transcripts was performed to identify variations, examine inconsistencies, and inform refinement to our coding scheme (XAE). The revised tables and memos served as the basis for the themes and the analytic framework described in the following results and depicted in Fig. 1. To corroborate our themes, a cross-thematic check was conducted subsequently, identifying variations and relevance of the themes across priority populations, including young adults, cis/trans women, racial/ethnic minorities, and individuals with suboptimal clinical engagement (XAE). Final themes were refined through feedback from our multi-disciplinary research team, their diverse areas of expertise provided insightful context to enhance our findings.

### Ethical Authorization

The University of California, San Francisco served as the Single Institutional Review Board (sIRB) for this multi-site study [38]. Written informed consent was obtained from all participants.

## Results

### Study Sample

Between August 2020 and July 2021, we conducted 72 interviews at Ward 86 (n = 35), IDP (n = 19), and at the Ponce Center (n = 18) (Table 1). This analysis focused on 70 interviews with participants who had not previously used LAI-ART; we excluded interviews with two individuals who had prior experience with LAI-ART due to their compassionate use access [34]. Participants’ age ranged from 22 to 75 years (median: 46); 20 (28%) were ciswomen, 5 (7%) transwomen, 32 (44%) Black/African American, and 25 (35%) Latinx. Many also reported challenges related to social determinants of health: 31 (43%) endorsed having a psychiatric diagnosis, 25 (35%) were experiencing homelessness/unstable housing, and 7 (10%) had methamphetamine or illicit opioid use in the last 30-days. In terms of care engagement, 17 (24%) were sub-optimally engaged in care, and of these, 8 (11%) had viral loads ≥ 200 copies/ml.

**Table 1:** Participant demographics and clinical characteristics (N = 72)

	N (%)
Age, median (min/max)	46 (22–75)
18–29 years	10 (14%)
30–49 years	29 (40%)
≥ 50 years	33 (46%)
Years living with HIV, median (IQR)	15 (7–24)
Racial/ethnic identity	
Black/African-American, Non-Latinx	32 (44%)
White, Non-Latinx	13 (18%)
Multiracial, Non-Latinx	02 (3%)
Latinx, all races	25 (35%)
Gender	
Cisgender female	20 (28%)
Cisgender male	46 (64%)
Transgender female	05 (7%)
Other	01 (1%)
Sexual minority orientation^b^	39 (56%)
Education^a,b^	
Less than HS	17 (24%)
High School Diploma/GED	16 (23%)
Some College/Technical School	26 (37%)
Post-Secondary Education	12 (17%)
Living situation	
Own/rent	47 (65%)
Unstably housed	10 (14%)
Experiencing homelessness (shelter/street)	15 (21%)
Financial situation^b^	
Struggling to survive	15 (22%)
Barely paying bills	16 (24%)
Have necessities	28 (42%)
Comfortable with extras	08 (12%)
Primary source of income^b^	
Cash income, wages	23 (34%)
Partner, family, friends	04 (6%)
Public benefits and payments	41 (60%)
Recent substance use (Meth or IDU)^b^	07 (10%)
Methamphetamine use, past 30 days	06 (8%)
Illicit opioid use, past 30 days	03 (4%)
Mental health diagnosis	31 (43%)
History of incarceration^b^	17 (24%)
Years on oral ART, median (IQR)	13 (6–20)
Currently taking Oral ART^b^	70 (99%)
Sub-optimal clinical engagement	17 (24%)
With viral non-suppression	08 (11%)

Sub-optimal clinical engagement: either two or more missed HIV primary care appointments in the past year or less than two HIV primary care appointments ≥ 90 days apart in the past year and a detectable viral load

Viral non-suppression: viral load results most proximal to the interview was > 200 copies/ml

^a^Totals may not equal 100% due to rounding to the nearest whole number

^b^Data missing: sexual orientation (n = 2), education (n = 1), financial situation (n = 5), source of income (n = 4), substance use (n = 1), history of incarceration (n = 2) and currently on ART (n = 1)

### LAI-ART Acceptability

Our analysis revealed three primary themes and two sub-themes regarding participant attitudes towards LAI-ART. In the first theme we characterize participants' responses to the idea of LAI-ART, which ranged from enthusiastic embrace to cautious hesitance to outright refusal. The second theme consists of four distinct attitudinal orientations that participants adopted when reflecting on and explaining why they embraced or refused the idea of LAI-ART. We labeled these attitudes as follows: the highly receptive *innovator orientation,* the cautiously optimistic *pragmatist orientation*, the ambivalent and uncertain *deliberator orientation*, and the resistant and doubtful *skeptic orientation.* These attitudinal orientations were driven by participants' personal beliefs, values, and prior experience with the healthcare system, and influenced how they responded to anticipated requirements and options for LAI-ART delivery, ultimately shaping their hypothetical willingness to uptake. The third theme describes the malleability of participant attitudinal orientations*,* which could shift as participants’ understanding of the treatment and its personal relevance evolved over the course of the interview. Two sub-themes emerged as important in considering engagement with LAI-ART information and heightened appreciation for its potential benefits. The first sub-theme highlights how discussion with their HIV provider was seen as instrumental for establishing personal relevance and facilitating uptake. The second sub-theme illustrates how HIV stigma, privacy concerns, and medical mistrust had varied impacts, sometimes facilitating and other times hindering personal relevance depending on the individual’s unique context. Figure 1 offers a visual representation of our findings.

#### LAI-ART Acceptability Spectrum

After presenting the education script, we asked participants for their initial impressions on LAI-ART. Most participants were enthusiastic, recognizing LAI-ART as a historic treatment innovation with the potential to significantly improve the health and quality of life for PWH. However, the salience and significance of these benefits varied among individuals.

##### LAI-ART Enthusiasm: “Goodbye pills, hello shots”

Participants who were initially enthusiastic about LAI-ART were highly receptive to switching to it, "If everything works out well and my body doesn’t resist it, I’m all in with it. … goodbye pills, hello shots." (30yrs, Black gay man, ATL) Acceptability was facilitated by an openness to novel therapies and a perception of low potential harm or risk of worsening health.

I learned about the shot in the buttocks once a month. I’m like, “Man, God must have heard my cries because that’s something that I can get with and I ain’t got to worry about all these freaking pills. (46yrs, Black heterosexual cisfemale, ATL)

Recognition of LAI-ART as a first-of-its-kind formulation appeared to ignite a sense of hope and optimism for the future of HIV medicine, including a potential cure. “I think it’s exciting and not just for the convenience of it, for just the fact that I think we’re getting closer to a cure.” (31, White heterosexual transwoman, SF) The sense of progress was especially impactful for those who have lived with HIV since the early days, often evoking gratitude. “What a wonderful thing science is—that we've evolved to, instead of me taking literally what was 20-plus pills a day, to now the possible evolution of—what are we looking at? Six injections a year?" (46yrs, Black gay man, ATL) LAI-ART’s potential to reduce the mental burden of an HIV diagnosis was also valued by participants. “I love [LAI-ART], no one likes knowing they have HIV. So, anything that allows me to think about it less would be perfect for me.” (43yrs, Black heterosexual transwoman, SF).

##### LAI-ART Hesitancy: “Makes me a little nervous”

Participants who were initially hesitant showed some interest but were unconvinced of LAI-ART’s personal relevance, particularly among individuals who were well engaged in care and virally suppressed on oral ART.

It's a very good option for some populations in the HIV world. But [for] people who are really on top of their meds – I only see my doctor once a year; you know?... I don't want to go to the clinic every month, that's too much. (31yrs, Black heterosexual Cisfemale, CHI)

Participants emphasized the importance of “seamless” and unobtrusive experiences. “It would be like, how seamless could you make it for me as a patient to, like, receive this medication and not make it too intrusive for me?" (22yrs, Latino gay man, SF) Others expressed hesitancy towards the inaugural formulation but might reconsider if longer-lasting options became available, “I’d want to get them up to like a three-month sort of thing… the less often [I] have to come in, the better.” (57yrs, White gay male, ATL) Participants expressing hesitancy voiced concerns about perceived loss of control over their treatment and dependence on others if they were to use LAI-ART. “I am in control of that pill…With [LAI-ART], I have to wait a month to come here where somebody else has the control of giving the injection. " (52yrs, White gay man, SF) Participants, especially those with multiple comorbid conditions, expressed concerns about risks of side effects, developing resistance, and disrupting their hard-earned pill-taking routine.

If you've only had HIV for a short time, you're not that nervous. But I had a T-cell of 13, which means I qualify as full-blown AIDS. It took me twelve years to get them over 400…so any loss in T-cells makes me a little nervous. (52yrs, White gay man, SF)

##### LAI-ART Refusal: “That's a deal-breaker for me."

Participants whose initial response to LAI-ART was a negative one readily identified dealbreakers that would prevent them from using it. Common dealbreakers included fear of needles and anticipated pain. "I am a big kid at heart. So, I just don't like pain. Two shots?! That's too much.” (27yrs, Black gay man, CHI) Others could not envision LAI-ART fitting into their daily routines. “If I forget something as simple as opening a bottle and taking a pill, imagine having to go somewhere at a certain time to get an injection…It would be more difficult for the injection to become a habit." (55yrs, Latino gay man, SF) Those who anticipated frequent or extended travel found coordinating recurring injection appointments unacceptable. “The thing that I value the most at this time is flexibility with my lifestyle. If the medication is going to keep me kind of tied to [this city] every month, I will not take it…that's a deal-breaker for me." (35yrs, White queer man, CHI) In a couple of instances, participants were concerned that LAI-ART might inadvertently encourage a lax attitude towards HIV care and treatment. They feared that the perceived sense of freedom associated with non-daily dosing could potentially lead to reduced diligence in managing their HIV. "That's good – but that's like making people more irresponsible. Because it's like people will start thinking they’re freer and freer…they're not going to remember to take care of themselves or that they have this disease.” (25yrs, Latinx heterosexual transwoman, SF).

#### Attitudinal Orientations Towards LAI-ART

We examined participants’ diverse reasoning and decision-making approaches and two groups emerged. Among those with favorable reactions, an important minority embraced novelty, but the majority adopted a pragmatic orientation. Among those with ambivalent or negative reactions, most embraced thoughtful deliberation, with select few adopting a skeptical orientation.

##### Innovator Orientation: “I feel good because I feel like I'm up to date”

Participants with an innovator orientation proactively sought out research to remain current with their treatment. “Staying vigilant with my HIV education and what I do with my body. It keeps me – I feel good because I feel like I'm up to date – the future of HIV." (43yrs, Black heterosexual transwoman, SF) The innovator orientation not only exemplified a pioneering spirit but also embodied a profound altruism, as these participants eagerly participated in research aimed at advancing treatment options. “When [my doctor] started talking to me about [LAI-ART], I definitely wanted to know about it, and I definitely wanted to participate in the study.” (46yrs, Black gay man, ATL) These participants viewed LAI-ART as the “next rung” in the evolution of HIV medicine and were committed to being part of its transformative journey. “I think [LAI-ART] is going to be the next rung in a new phase of HIV drugs. I want to be a part of the next group.” (65yrs, multi-racial gay man, SF).

##### Pragmatist Orientation: “My decision is because it makes everything easy”

Participants with a pragmatist orientation, the most common, expressed interest in trying new therapies for more practical management of their health and to improve their quality of life.

Interviewer: How might you go about making this decision about LAI-ART?

Participant: “Well, my decision is because it makes everything easy for you...I think it's more practical - I think it's more effective to have a shot every four weeks, so you don't miss any pills…Having the injection every four weeks gives you better control...you can rest assured that for the next [month] the medication is working…" (64yrs, Latino bisexual man, SF)

The primary benefit most frequently mentioned was the desire to alleviate the burden of taking pills. "An injectable once a month is just one less pill to take. I have high blood pressure and high cholesterol and I’m having to take a pill for that, so one less pill every day is great." (46yrs, Black gay man, ATL) The psychological burden of adhering to a strict regimen of daily oral medication was a significant motivator that frequently outweighed concerns about physical discomfort or logistical challenges. “There is a level of stress and anxiety around maintaining the medications and understanding that you really don’t want to allow resistance to happen.” (30yrs, White gay man, SF) These participants valued the convenience of LAI-ART, viewing it as reducing the psychological burden of daily pill-taking and offering a more practical approach to HIV self-management “The biggest thing is to get it monthly vs. having to do it every day. That would be the biggest thing that makes me want to start, to forego having to pop a pill every morning…” (57yrs, multi-racial gay man, ATL).

##### Deliberator Orientation: “I’m not saying no and I’m not saying yes”

Participants with a deliberative orientation took a cautious approach to LAI-ART, valuing a thorough consideration process to weigh its benefits and risks. They prioritized in-depth discussions with trusted individuals to facilitate a comprehensive understanding of LAI-ART and its personal relevance. “I’m not saying no and I’m not saying yes to it, but it’s just something that I have to think about over time.” (28yrs, Black heterosexual transwoman, ATL) Continuous discussions with their HIV provider were commonly expressed as essential to solidifying their acceptability of LAI-ART.

The injection would be something that would need to be discussed and it would be a matter of looking at it in more detail over several months with my doctor…Every time I have an appointment, we're going to talk about it, because that would be good. (61yrs, Latino man, SF)

Additionally, they stated they would actively seek diverse perspectives to inform their decision-making, often through independent research and consultations with other healthcare professionals. "I would be in contact with my previous infectious disease doctor…I would get with the infectious disease physician at Presbyterian also. If this is for me, I would consider it…But I would do some research.” (75yrs, Black bisexual man, CHI) These participants wanted a comprehensive understanding of LAI-ART and stated they would make informed decisions by acquiring knowledge and insights from a range of sources, including clinical trials and firsthand accounts.

It’s relatively new, so a part of me wants to wait six months or a little bit longer, seeing its efficacy, how it’s working, what people are saying, getting their opinion or their review on how the injection feels, how it’s working before I jump on it. (52yrs, White gay man, SF)

##### Skeptical Orientation: “I’d need to answer that with a question: What for?”

Participants with a skeptical orientation were doubtful and resistant to LAI-ART, often citing concerns about its efficacy, safety or necessity. "What for? What do I have to gain from the injection that the pill I'm taking now doesn't provide?" (55yrs, Latino gay man, SF) The novelty of LAI-ART raised concerns about long-term side-effects and being seen as test subjects, reflecting deep-seated mistrust in medical research. “I won't be a guinea pig…You're not going to end up dissecting me because your drugs killed me.” (59yrs, White gay man, ATL) They viewed LAI-ART as a more invasive option that implied worsening health. “I don't think it would be for me. I'm just so used to being on the [pill]. And I don't think my CD4 is that low where it has to be, you know, used with that type of medicine." (27yrs, Black gay man, CHI) They offered decisions based on security, comfort, and confidence in their current treatment. “I’m fine with the pills. I would be fine not to take them. Again, a cure would be grand. And then, I could stop taking these pills… But, no, until then, I have to continue taking these pills." (59yrs, White gay man, ATL) Skepticism also arose from the perception that the allocation of resources to develop new treatment options could divert attention and funding away from HIV cure research. “What people want is the vaccine. They don’t want implants. Forget about those things…if they already have a way to control it, how is it that they don’t have a way to put an end to it?” (53yrs, Latino bisexual man, SF).

#### Attitude Malleability with Increased Understanding and Perceived Personal Relevance

As participants processed information about LAI-ART's anticipated requirements and delivery options, attitudes generally became more favorable over the course of the interview and often caused shifts in attitudinal orientations (see Supplementary Information). The most common shift occurred among skeptics who later adopted a deliberative or pragmatist orientation. Enthusiastic innovators were an exception, expressing strong interest early and consistently. However, a multitude of factors beyond information often played a pivotal role in shaping personal relevance and acceptability of LAI-ART.

##### LAI-ART Perceptions Can Evolve: “Knowing what I know now…”

We examined how delivery options and anticipated requirements, such as pre-existing viral suppression, an oral lead-in, and oral ART bridging, deepened participants' understanding and appreciation of LAI-ART's benefits. The availability of an oral lead-in phase reassured some participants, allowing them to envision a path for gradually adopting LAI-ART and addressing safety and efficacy concerns on their terms. “It's more comfortable, to be honest, just understanding that you were getting a feel of the medication and how it works, versus going straight to the shot and living a whole different life.” (23yrs, Black bisexual man, CHI) Participants who initially perceived LAI-ART as inflexible, saw the potential for oral bridging, or temporary use of oral ART when late for an injection, as a way to maintain flexibility and control over their treatment.

You mean, I’m not tied down exactly to the 30 days. If people go on vacation and they have to get this medication there is a safeguard, an option to take the pills…I think that would be good so people can feel more in control of their lives. (22yrs, Latino gay man, SF)

On the other hand, a minority of participants expressed additional concerns as they reflected on the real-world implications of using LAI-ART including increased time off work, extra time and financial resources for commuting to and from the clinic, potential out-of-pocket cost related to insurance copays, and approval for patients with stable viral suppression on oral ART (see Supplementary Information).

##### The Crucial Role of HIV Providers in LAI-ART Decision-Making and Uptake

The majority of participants had a high degree of trust in their HIV providers and consistently emphasized the importance of consulting with them to establish personal relevance (see Supplementary Information). Specifically, participants expressed a desire for personalized education on LAI-ART and stressed that shared decision-making with their healthcare provider as essential for considering its uptake.

I would expect my doctor to sit down with me and say, here's the drugs, here's what they do. Here's what's different about these drugs versus what you're on. There should be no break in staying undetectable…So, as long as there were very mild side effects, I'd have no trouble switching. (61yrs, White gay man, SF)

However, participants’ preferences regarding their level of involvement in the decision varied, some suggested they would be bringing up the topic themselves, while others expressed confidently that their provider would initiate the conversation if deemed right for them.

##### HIV Stigma, Privacy Concerns, and Medical Mistrust Impact Personal Relevance

We explored how participants' perceptions of HIV stigma, privacy concerns, and medical mistrust influenced the evolving understanding and personal relevance of LAI-ART. These factors had diverse effects, at times facilitating and at other times impeding acceptability, depending on each individual's unique circumstances. For many, the initial attraction of LAI-ART lay in its potential to alleviate concerns about unintentional HIV status disclosure. "Being able to not have pills around. That gives me wanting to do it just to be able to not have my medicine in my house no more where only me and my doctor know what's going on.” (52yrs, White gay man, SF) However, upon deeper contemplation, some of these same participants began to express concerns that increased clinic visits might serve as a reminder of their stigmatized HIV status. Others expressed concerns that increased healthcare interactions and potential injection “marks” might expose their HIV status accidentally. “You know, like is it the same spot?…Is it going to cause a mark? That’s kind of what I was worried about.” (33yrs, Black bisexual man, CHI).

Prior interactions with providers and institutions played a significant role in shaping personal relevance and acceptability of LAI-ART. Those expressing mistrust of healthcare systems, pharmaceuticals corporations or the government often extended that mistrust to LAI-ART, “It's a lot of stuff on the news, like, medicines occasionally don't agree with people. I don't want [more] health issues.” (28yrs, Black heterosexual transwoman, ATL) This mistrust was stronger among those reporting a history of side-effects and adverse reactions on oral ART. Additionally, some participants expressed a sense of mistrust in the healthcare system's priorities, with particular criticism directed at implementation policies, notably the delayed introduction of novel treatments in underserved areas. This mistrust significantly impacted their perception of LAI-ART’s relevance and acceptability, “I guarantee you the north side is going to get it first, and then the south side. They always make [us] second. Always…That’s just not fair.” (33yrs, Black heterosexual cisfemale, CHI).

### LAI-ART Attitudes Among Priority Populations

We enhance theme validity by confirming relevance and examining variations among priority populations. These themes remained consistent across young adults, cis/trans women, racial/ethnic minorities, and individuals with suboptimal clinical engagement, as illustrated in supplementary information.

#### LAI-ART Attitudes by Priority Populations

Young adults (n = 10) perceived LAI-ART as a means to improve adherence but expressed concerns about needles, additional clinic visits, and approval with stable virologic suppression. Many, particularly those of minority backgrounds, viewed these barriers as dealbreakers. Learning about the oral lead-in and oral bridging options often led to greater acceptability.

Ciswomen (n = 20) acknowledged LAI-ART’s potential adherence benefits but expressed concerns about broader impacts if the treatment failed. Some wanted to discuss the option with trusted peers and family in addition to their HIV provider. Frequent injection visits and managing side-effects were common dealbreakers, but longer treatment duration could prompt reconsideration. In hypothetical pregnancy scenarios, infant safety was often prioritized.

Transgender women (n = 5) voiced both excitement and caution about LAI-ART. Many acknowledged the potential psychosocial benefits, including relief from the mental burden or the ability to create psychological distance from HIV. Experience with injectable hormone therapy often alleviated concerns related to effectiveness, injection pain, and routinizing injections, however, worries persisted about side-effects, pain tolerance, and perceived complexity.

Black/African-American participants (n = 32) viewed LAI-ART as a means to improve adherence and gain greater control over HIV disclosure, reflecting a consistent emphasis on health privacy. Those reporting narratives of having overcome HIV stigma and being more open about their status were more likely to assume a deliberative or skeptical orientation, particularly those comfortable with oral ART, well-engaged, and virally suppressed. Potential obstacles like transportation, additional out of pocket costs, and side-effects were commonly mentioned.

Latinx participants (n = 25) were excited about LAI-ART as a treatment option. However, those who faced systemic stigma in their countries of birth and here in the U.S. often experienced cognitive dissonance when visiting the HIV clinic, as it reminded them of their stigmatized disease and made them feel disconnected. Trust in their medical provider alleviated worries about increased clinic visits for injections, as they often considered them as family and valued their expertise.

Participants with suboptimal clinical engagement (n = 17) generally expressed enthusiasm and pragmatism about LAI-ART, emphasizing its adherence and privacy benefits. Additionally, some who were not currently virally suppressed (n = 8) valued the prerequisite of stable viral suppression before initiation, seeing it as facilitating a smooth transition and an opportunity to contribute to their treatment. However, a few expressed demotivation or noted a paradoxical quality to the stable viral suppression requirement.

## Discussion

In a multi-site qualitative study of LAI-ART acceptability conducted just prior to its clinical availability, we found that while initial reactions varied, most deemed LAI-ART an acceptable alternative to daily oral medication. We characterized four distinct attitudes—the innovator orientation, pragmatist orientation, deliberator orientation, and skeptic orientation—however, these attitudes were not static. Rather, attitudes were remarkably malleable as participants considered and processed information about LAI-ART. Central to this transformation was the pivotal role of HIV providers, with medical consultation viewed as essential for determining the personal relevance of LAI-ART for their lives. Conversely, HIV stigma, privacy concerns, and medical mistrust had varied impacts, sometimes facilitating and other times hindering participants’ interest in and acceptability of LAI-ART depending on their unique context. These findings were consistent across priority populations, including young adults, cis/trans women, racial/ethnic minorities, and sub-optimally engaged participants.

Our findings align with Carillon et al. (2020), who identified significant ambivalence towards LAI-ART among PWH and highlighted how some may perceive a potential decrease in autonomy with LAI-ART use [39]. Similarities in our findings, specifically related to hesitancy as an orientation and the overall malleability of attitudes, prompt us to join them in problematizing the simple acceptable/not acceptable binary and temper the reported "high acceptability" of LAI-ART in clinical trials [40, 41].

Further, we draw relevance from Koester et al.’s (2021) qualitative study of PrEP uptake, which found that the shift from awareness to uptake rarely occurs during initial PrEP educational encounters, instead, it is an evolving process arising from repeated exposure to diverse information, sources, and channels [42]. A key point of convergence is the role of personal relevance, encompassing alignment with individual medical needs and the potential to enhance the quality of life and overall well-being, in shaping readiness to uptake. In both studies, individuals who found personal relevance in the novel treatments were prompted to investigate the topic further, occasionally resulting in a transformative shift in perspective and greater appreciation for the potential benefits for their lives [42].

Consistently across our three studies, medical providers emerge as playing a crucial role in affirming the personal relevance of treatments and facilitating their uptake [39, 42]. This underscores the inter-subjective nature of acceptability, emphasizing its dynamic development through interpersonal interactions and mutual understanding [43, 44], contrasting with traditional perceptions that prioritize retention of factual information as a key precursor of behavior change.

A core finding of our study is that initial perceptions of LAI-ART acceptability are dynamic, not static or fixed. Indeed, our research helps elucidate *how* PWH may reason through their thoughts and considerations regarding LAI-ART. This journey unfolds in stages: forming initial impressions, articulating attitudinal orientations, and occasionally experiencing transformative shifts in attitudes with new information. This perspective reaffirms the idea of acceptability raised in recent HIV literature as an emergent interactional accomplishment [42], influenced by diverse contextual factors [16, 17], and significantly shaped by the clinical encounter and the patient-provider relationship [39].

Our findings are also consistent with other literature that explores the perspectives of PWH on LAI-ART. Our study found facilitators of uptake similar to existing qualitative research, notably the recent work by Gonzales Rodriguez et al. [45] such as perceived ease/convenience of adherence and privacy benefits, as well as concerns related to effectiveness, side effects, costs, and increased clinic visits [23, 24, 28, 29, 45]. However, our study goes a step further, past categorization of facilitators and barriers, to highlight the process by which participants weighed these pros and cons. Some participants remained steadfast in a point of view while others shifted perspective as they continued to reflect on the benefits and potential downsides of switching from daily oral pills to monthly injections. Our analysis makes a novel contribution to the literature by proposing a model that emphasizes this information processing and the patterned responses it produces, which has implications for patient education and counseling. Our findings suggest that in addition to information, participants want provider discussion with shared decision-making. Encouragingly, this work is already underway, Philbin et al. [46] are developing a decision-making aid to help women choose between oral ART and LAI-ART. However, further research is needed to assess the durability of choices made with these tools and the impact of decision aids on healthcare systems.

Recognizing that the aim isn't to persuade all people with HIV to switch to LAI-ART, we propose the following recommendations to enhance implementation efforts: Safeguard time for comprehensive and ongoing discussion between patients and trusted healthcare providers about LAI-ART. When a trusted provider is unavailable, adopting a person-centered approach that allows for a broader exploration of motivations and concerns, beyond those directly related to HIV treatment and prevention, may be beneficial. Proactively address HIV stigma, privacy concerns, and medical mistrust, as these factors can obscure personal relevance. Tailored messaging that addresses these barriers may promote recognition of the potential benefits of LAI-ART. While some individuals may readily see the advantages, others may require more time and increased trust in both providers and healthcare systems before proceeding.

Our study's findings may have limited transferability to non-urban or less diverse clinical settings. Even though our sampling emphasized perspectives of individuals sub-optimally engaged in care, clinic-based recruitment did not facilitate gathering perspectives of those out of care, where LAI-ART could potentially serve as a tool to re-engage patients in care. In addition, ensuring the general high-level themes were upheld across sub-populations necessarily resulted in less focus on the nuanced findings that may pertain exclusively to these groups. As such, more research is warranted to delve comprehensively into the specificity of attitudes within each group, and many groups may benefit from dedicated explorations with larger sample sizes. Finally, the study relied on prospective intentions, which may or may not align with actual decision-making about LAI-ART. Moreover, despite employing a semi-structured interview guide, an education script, and experienced interviewers, the possibility of differences in style of interviewer questioning and probing exists. Still, we believe our findings provide a useful starting point for approaching future evaluation efforts.

In summary, we found that LAI-ART acceptability is a malleable construct shaped by ongoing discourse, with distinct attitudinal orientations that can shift as understanding deepens, and that acceptability is profoundly impacted by provider recommendation as well as the potential contribution of stigma, privacy concerns, and medical mistrust.

### Supplementary Information

Below is the link to the electronic supplementary material.

Supplementary file1 (PDF 111 KB)

Supplementary file2 (PDF 134 KB)

## Data Availability

All data generated and used in this research was independently collected by the study team. Data in this manuscript will not be publicly accessible to protect individual privacy and comply with Institutional Review Board requirements.
