# Supplementary material for: Acceptability of Long-Acting Injectable Antiretroviral Therapy Among People with HIV Receiving Care at Three Ryan White Funded Clinics in the United States
Source: AIDS Behav. 2024 Apr 10;28(7):2226–38. doi: 10.1007/s10461-024-04315-0 (PMC11199206; doi:10.1007/s10461-024-04315-0)
Supplement: Supplementary file 1 — Supplementary file1 (PDF 111 KB) [file 10461_2024_4315_MOESM1_ESM.pdf]

LAI-ART Attitudes/Preferences
Patient Interview Guide

Opening/Warm-Up:

1. Tell me how your day is going so far. What have you been up to?
2. What do you have planned for the rest of the day after we finish here?
3. How typical would you say today is for you?

Current Health Assessments:

4. How would you describe your health these days?
    a. What do you take care of your health these days?
    b. What, if anything, could the clinic do to help you better manage your health?
    c. How has COVID-19 impacted your day-to-day routine?

Let's switch topics. I'd like to ask you some questions about your experiences with XX clinic.

Engagement in HIV Care:

5. Tell me about your experience at [Name of Clinic]?
    a. How long have you been a patient here?
    b. How did you get connected to [Name of Clinic]?

[Note, for well-engaged patients, Q6 is okay to ask next, if they are not well-engaged, we will want to consider moving this down.]

6. In general, how easy or difficult is it for you to keep your appointments?
    Potential probe:
    a. What feelings come to mind when you think about coming in for your appointment?]
    b. What are some of the reasons why you miss appointments?
    c. How important is it for you to be reminded about your appointment, for example, the day before with a phone call or text. [not sure if this is a practice at each site?]
    d. In terms of getting to your appointments, have you ever received bus tokens or taxi voucher paratransit) to make sure you are able to keep your appointments?

7. Have you had an appointment since the shelter in place order was implemented? Tell me about it
    a. Did you come in in-person to see your doctor? If so, how did you feel about that?
    b. Have you had an appointment done over the phone?
    c. If patient has had a phone visit: How does a phone appointment compare to an in-person appointment? Which do you prefer? What makes you prefer X over Y?

Relationship To HIV Provider:

8. Tell me about your relationship with your HIV provider here at the clinic.

   a. [Potential probe:] Some patients are very comfortable and open when they talk with their provider. But other patients do not feel completely comfortable talking with their provider. What's it like for you to talk with your provider? Probe for being open, feeling comfortable, able to ask questions, understand responses and explanations from provider.

9. What about when it comes to making decisions about your care, for example, starting a new medication. How does that work?
   a. Other than your provider, who or what other factors come into play when you are making decision about your health?

<u>**Experience With/Attitudes Towards Oral ART**</u>:

10. Tell me about your experience with HIV medications.
    a. ON ART: Some people have no trouble remembering to take their medications every day and others might have difficulty remembering to take their medications, how would you describe your situation with taking your meds?
       i. [Potential probe:] Out of curiosity, where do you store your medications?
       ii. I'd like to hear about how you feel about taking anti-retroviral therapy every day.
    b. IF NOT CURRENTLY ON ART: What are some of your concerns?
       i. Can you imagine a situation where you would feel that treatment was right for you? If so, tell me more about that

11. I'd like to hear about a time when you may have stopped taking your HIV meds for longer than a few days. Tell me about that.
    a. What was happening in your life at that time?
    b. Did you discuss this with your provider before stopping? Why or why not?
    c. Did you discuss this with your provider after the fact? What did they say?
    d. For a lot of people, using alcohol or other substances or drugs can get in the way of taking medicine and/or coming to appointments. Has this been an issue for you? If so, tell me about it.

<u>**Awareness of Modern LA ART Technology:**</u>

12. So, we talked about it a little, but did you know about LAI-ART before this study?
    a. If so, where did you hear about it? What do you know about it?
    b. [If have heard about it:] How would you define it?
    c. Have you had any personal experience or know someone who had personal experience with long-acting medications? If so, tell me about that.

<u>**** [Education Script Shared With & Read To Participant] ****</u>

READ TO PARTICIPANT: Long-acting HIV medications are antiretroviral medications that stay in the body over a longer period of time when compared to a daily oral pills. The long-acting

antiretrovirals that are furthest along in development and likely to be approved soonest are two medications called cabotegravir and rilpivirine given as two injections in the butt muscle every four weeks.

It is probably not quite as intense as a penicillin shot for syphilis, but more than a flu shot in the arm. Some patients may have resistance in their virus that means that these particular long-acting HIV medications (cabotegravir and rilpivirine) are not an option for them.

Another key point is that currently, to start injectable cabotegravir and rilpivirine, you have to be virally suppressed. It is to say, one must have an undetectable viral load.

Finally, it's very important that people come for their injections regularly – if they are going to stop, then they have to go back on pills, otherwise they might develop resistance because the long-acting drugs can stay in the body for a long time at levels that are not effective as treatment against HIV.

Really need to emphasize that LAI-ART may not be an option for some patients based on whether their virus has resistance to certain classes of medications (NNTRI, integrase)

Considering LAI-ART:

13. So, now that I have provided you with more information. What do you think of LA HIV Treatment?

    Potential Probes:

    a. What is appealing to you about LAI-ART?
    b. What is unappealing?
    c. Do you think LAI-ART would be right for you?

[Ask questions in Blue to patients stating that LAI-ART **IS OR MAY BE RIGHT FOR THEM**]
[Ask Questions in Orange Q30 to patients stating that LAI-ART is **NOT RIGHT FOR THEM**]

14. [ONLY ASK: Patients stating that LAI-ART IS or may be right for them.] What would it take to make you feel comfortable using LAI-ART?

    Potential Probes:

    a. What more information would you like to have about LAI-ART?
    b. What might get in the way of using LAI-ART?
    c. What would help you take LAI-ART?
    d. Describe a time in your life when LAI-ART would not have been right for you.

15. What would be the most crucial factors for you in deciding to use LAI-ART? What are your concerns? (Probes: efficacy; side effects; needles; pain; number of injections; intimacy of gluteal injection – who gives shot; having to come to clinic; cost, etc.)

    a. Have you ever had a negative experience getting an injection in the past?

b. Some patients are sore around the injection site. How much of a concern is this for you?
c. For individuals who express a fear of needles: Tell me a more about what makes you feel afraid. (Probe: How does this affect how you feel about LAI-ART?)

16. ***[For women only]***: LAI-ART has not been studied in pregnancy, though in general many HIV medicines are considered safe in pregnancy. Would thinking about becoming pregnant impact your decision to take LAI-ART?

17. [***For people who have referenced a social support network***]: How do you think the people in your life who are important to you would respond to your taking LAI-ART? Probe: Partner, family, friends, etc.

Prescription Requirements:

18. LAI-ART is currently intended to be used after people become undetectable on an oral regimen first. Then, they take two medications that are in the injections in oral form (two pills once a day) for 30 days before getting their first injection. What do you think about that requirement? (Probes: What would help you with the oral phase? What would make it difficult?)

19. It also may be possible to proceed directly with the injection without taking the oral regimen first. What do you think about this option? Which would you prefer?

20. If you are going to be more than a week late for your injection, you need to start taking HIV medication in the form of a daily pill again to not develop resistance. Therefore, when someone starts LAI-ART they are given a supply of pills to keep on hand in case they can't get to the next injection on time. What do you think about this requirement? How does this impact – if at all – your feelings about LAI-ART?

Optimizing Delivery of LAI-ART:

21. Let's say you could get this injection every month at a time and place of your choosing – a time and a place that would ensure you got the shot every month. Take a minute to imagine that and consider it, and then tell me about it.
    a. Where would you be willing to receive LAI-ART – e.g. clinic, home, pharmacy, community-based organization (methadone clinic), mobile van/clinic? Which would you prefer? Why? Is there another location you would prefer that I haven't mentioned?
    b. How does COVID affect your preference for where you would like to receive the shot every month?

22. How would you feel about being referred to another provider that is dedicated to the delivery of this medication? What about this approach is appealing? What is unappealing?

Other Concrete Suggestions:

23. First, how would you want to be reminded of upcoming appointment for shot/injection? (Probe: text, call, need a person to go with?) Who would be the best person to remind you about the appointment?

24. Would you prefer to have a scheduled appointment or be able to drop-in? Would it be important to you to see your provider at the time of the appointment? Why/why not? [Potential probe: length of injection appointment, wait times]

25. Who would you prefer to give you the LAI-ART injection? (Probes: Provider, nurse, pharmacist, etc. How important would it be for the same person to give you the injection each time?)

26. Can you think of things that might get in the way of you being able to continue to come in for your appointments or get your injections?
    a. How do you feel about coming in every month for an injection?
    b. What would be some of the challenges and can you think of any benefits you would get from coming in more often?

27. [***For individuals with a history of substance use***:] Imagine a time when you are back to using [insert substance use] and you miss an appointment- how would this impact how you feel about receiving services related to LAI-ART in the future? (Probe: Shame-cycle)

    Probe: How would you feel about clinic staff or members of your healthcare team reaching out to you if you were back in your addiction? What would be the most successful approach?

28. If you missed the injection appointment: How would you like to be followed-up with? (Probe: text, call, in-person visit). Who would you want to reach out to you (e.g., nurse, doctor, etc.)?

29. [ONLY ASK: Patients stating LAI is NOT right for them] What is appealing to you about the oral pill over the injectable?
    a. Potential probes: Routine, independence

30. You said you don’t think long-acting is right for you, tell me more about that.
    a. Potential probes: Increased visit frequency, needles, pain, gluteal injection
    b. For individuals who express a fear of needles: Tell me a little more about what makes you feel afraid.

31. Was there a time when LAI-ART would have been right for you? Tell me about it.

32. What about a time in the future – how likely would you be to consider long-acting in the future?
    a. Can you think of something you could change that would make LAI-ART more appealing?
    b. If there is any, what more information would like to have about LAI-ART?

33. If LAI-ART is an option for you based on your treatment history, how likely do you think you are to use it when it becomes available? (Probe: Can you tell me about how you might make the decision to use it?)

Now I'd like to ask you about some other long-acting medications that might be available in the future.

Future LA Technologies

34. Scientists are studying LAI-ART that you can inject by yourself at home, similar to insulin for diabetes. What do you think about this idea?
    a. Another option is that a partner or a friend could administer LAI-ART. What do you think about this idea? Which do you prefer?

35. LAI-ART has been studied with being given every 4 weeks, though scientists are now studying every 8 weeks. What would the best time between doses of LAI-ART for you? Why?
    a. (Probe: If doses are too far apart, some people feel it would be a challenge to remember. What do you think about that?)

36. In addition to injections (these are the ones closest to being available), there are other long acting treatments (like an implant) that could last up to 6 months of a year. What do you think about this idea?

Closing Questions

37. We have talked about many aspects of treatment and care for HIV. Is there anything else you think I should know about how you feel about long-acting HIV medications?

38. Are there any last thoughts you would like to share? Is there any topic that you thought we would discuss that I didn't ask you about?

39. Do you have any questions for me? What are your thoughts about this interview?
