# Supplementary material for: Acceptability of Long-Acting Injectable Antiretroviral Therapy Among People with HIV Receiving Care at Three Ryan White Funded Clinics in the United States
Source: AIDS Behav. 2024 Apr 10;28(7):2226–38. doi: 10.1007/s10461-024-04315-0 (PMC11199206; doi:10.1007/s10461-024-04315-0)
Supplement: Supplementary file 2 — Supplementary file2 (PDF 134 KB) [file 10461_2024_4315_MOESM2_ESM.pdf]

	Initial Reactions	Facts & Requirements	Decision-Making & Medical Professionals	Medical (Mis)trust	HIV Stigma & Privacy Concerns	Uptake Intentions
**Young Adults**						
Case Study 1<br><br>22YO Latino Gay Male SF<br><br>DELIBERATOR TO PRAGMATIC	HESITANT- DELIBERATOR: “If it's somewhat closer to like penicillin, then I'd be kind of like be hesitant to take it. But you know, there are tradeoffs you have to make if you're not going to take the pills. I mean, two days of discomfort, I guess, isn’t terribly bad. You know?"	VISIT SCHEDULE: “[LAI-ART is] probably not [right for me] just because I don't think I would be willing to make time to go to the clinic every month.”<br><br>BRIDGE PILLS: “I could see myself on this…I'm not tied to go in on like the 30th day… And I think [bridging pills] would be a good thing, so people can feel more in control of things.”	PAIN TOLERANCE: “If it were a little bothersome for a little while, I would consider going on the injection if it was compatible with the virus type I have."<br><br>SECURING PRESCRIPTION: “He'll be like, “that's okay”. And give me options to resolve the problem or refer me elsewhere for help. [My doctor is] very professional and nice."	TRUST-HIV SCIENCE: “I'm not going to let myself die, so take the meds and see how it goes. But also, I think it's because I live with a roommate who is HIV-positive, so it's like I see him day to day and it's like he's alive. So, for me I think it kind of gave me a perspective… people don't necessarily die from this very often anymore."	HIV STIGMA: “I think my age group is more open to telling people they're taking medication, for like depression or something. It's almost a joke, "Oh, I'm taking this."…I also don't flaunt it, either. But if someone came into my room and saw my medication, I wouldn’t have a problem telling them about it."	PRAGMATIC: "I think it's very likely - I would highly consider it if it were available to me. Because knowing what I know now from the [interview], it seems a little bit more convenient than the pills…My only issue would be like, how long it would take to get the injection each month.”
Case Study 2<br><br>23YO Black Bisexual Male CHI<br><br>PRAGMATIC TO SKEPTICAL	ENTHUSIASTIC-PRAGMATIC: “I mean I didn’t really have any problems with it…I know that I'm very consistent with places and people that hold me accountable. So, I know that I would be consistent with it.”	INJECTION PAIN: “I can deal with shots – don’t get me wrong –they hurt. But it's that pain, I don't know if I want it in my butt. You know?"<br><br>ELIGIBILITY: "I'm very close to the load I need to be. Maybe, I can bring up this opportunity to [my doctor] in a few months or so, when I'm at that level."	COMFORT WITH ORAL ART: “I learned how everything works, when I got in the hospital - why the pill works the way it does. And I'm okay with that."<br><br>SECURING PRESCRIPTION: “I would just let [my doctor] know I'm over taking the pill and I want to have a different experience with [LAI-ART].”	TRUST IN PROVIDER: “Honestly, I love [my doctor]. He's a very down-to-earth person. He's one of the first doctors that asked about my personal life. I feel like he's someone who wants to really know [why am I so strong.]”	HIV STIGMA: “I've already been through a lot. So, this is kind of reminding myself that I'm not normal and that I won't ever be normal. So, it's like I already established what the situation was and accepted what it was for what it is."	SKEPTICAL: “I don’t really see it being the priority right now. Just because I'm still kind of recovering from so much.”
**Cis-Women**						
Case Study 3<br><br>40YO Latina Heterosexual Ciswoman SF<br><br>PRAGMATIC	ENTHUSIASTIC-PRAGMATIC: “I think it would be easier not to be taking medication, and mostly it's enough for me that instead of taking medication every day, it would mean coming in only once every two months, every eight weeks. That would be great.”	ORAL BRIDGE: “Sometimes you can't get there on time or you can't make it…If you couldn't make it because of an emergency, you have the pills. That would be good."<br><br>PREGNANCY RISKS: “If I were thinking of getting pregnant, then I would have doubts, because I would be thinking there might be risk of transmission to the baby.”	PILL BURDEN RELIEF: “It would be like, “I'm going to the clinic for the injection,” and not taking pills depends on me doing that…It also takes away that sadness, “Oh, I have to take my pill.” I wouldn't have that hanging over me anymore."<br><br>SECURING PRESCRIPTION: “If the doctor said, “Oh, look, this injection is good. Do you want to try it?” “Oh, of course.” If it were available, of course!”	MISTRUST: “It does scare me a little bit because of the side-effects and that it might not work, or it might reactivate the virus [HIV] back."<br><br>MISTRUST: “If I'm not 100% healthy…I may get sick from COVID, which I do not want. I would be afraid that my immune system might not be strong because of [LAI-ART]. Do you understand?”	HIV STIGMA: “You have to remember its for HIV. It's like you get frustrated, but at the same time, it's for my own good…deep breath, ‘okay, I have to do this’…It would help a lot, it really would."<br><br>PRIVACY: “No one knows of my problem, so I have to be well. My daughter doesn't know, and neither do my parents. So, I need to always be 100% well."	PRAGMATIC: "It’s all fine. I mean, the medication is here, and yes, I really would like to get the inject injection if it works. I would really be very interested."

Case Study 4<br><br>48YO Black Heterosexual Ciswoman CHI<br><br>PRAGMATIC TO DELIBERATOR	ENTHUSIASTIC-PRAGMATIC: “I always talk to [my doctor] about I hope, one day, they come up with something where I can get a shot and don't have to take those pills…I talked to [my doctor] last week and he said, "Well, I think your prayers have been answered. “	LEAD-IN: “I would prefer the 30 days. When I try new pills, I usually have an upset stomach for two days, but the symptoms subside real quick…I think the pills will be pretty much the same effect as the injection.”<br><br>BRIDGE PILLS: “I know things could happen – [that’s why you need] plan, A, B, and C...If I missed a shot, I would take the pill immediately because this is my safety net. So, I know to go straight to it.”	FAMILY VALIDATION: “What would make me more comfortable? I know they can't promise I won't have side effects because there are many. But I'm going home today and I'm going to talk to my family about this.”<br><br>SECURING PRESCRIPTION: “When I make decisions for my care that I'm really not sure about, I always ask questions. I never go with the flow. I make sure that I get information that is needed.”	MISTRUST: Like this coronavirus vaccine - I really want to take it, but then I think about all these allergies. Just, every time I try something new, there is something."<br><br>MISTRUST: “I'd be kind of skeptical about like taking when it's something new. I mean I have allergies very bad. Anything I try new, it is a bad outcome for me. I mean it be a bad outcome for me."	HIV STIGMA: “When I was first diagnosed, I was not taking my pills. So, when I transferred - [my doctor] said, "You have to do this. You can live a long life if you take the pills.” I promised him I would do better. And I’ve kept that promise."<br><br>PRIVACY: “My last clinic wasn’t very confidential. If I brought someone, they never asked, "Can we talk in front of this person?" I needed a clinic where I could have confidentiality and know it's important to them too."	DELIBERATOR: “This is something I want to discuss with my family, you know, to get their opinion, you know, and what they think, you know, their overall thinking and how can they be supportive to me, making sure that this is something that I stay on top of as well. You know?”
**Trans-Women**						
Case Study 5<br><br>43YO Black Heterosexual Transwoman SF<br><br>INNOVATOR	ENTHUSIASTIC-INNOVATOR: “It's just kind of mind-blowing! I always thought and wished and hoped in the back of my mind, but I didn't know that it was a definite thing… I'm ready to give it a try – eager to give it a try."<br><br>EXISTING INJECTION ROUTINE: “I do well with injectables. I'm very sure 99.9% sure I can keep a regular - one appointment every month."	INJECTION PAIN: “I'm more comfortable with the butt, just because I have more tissue down there. I'm also thinking the needle has to be bigger, but my hormone needle is pretty big too, so I'm okay with that."<br><br>LEAD-IN: “I think in that [first] month they need to have the results of that medication once it's inside you - it makes total sense to me."	OTHER INJECTIONS: "I get hormone injections twice a month. Every two weeks."<br><br>INJECTION EXPERIENCED: “If I came the same day, I would choose one for [LAI-ART] and the other buttocks for the estradiol… if they couldn't do both the same day, that's fine. That's not a concern."	TRUST IN PROVIDER: “He's really supportive, I've had some issues and he's just really supportive with anything I need from him... I'm grateful for him – because I know I'm going to be honest with him with what I know. He'll be honest with me with what he knows, and then, boom, I make a decision."	HIV STIGMA: “My family, my close friends know...Honestly, none of my partners know. I've never disclosed to them."<br><br>RESILIENCE NARRATIVE: “Those shingles really shook me…That let me know you are HIV-positive, and it's affecting your body, and it is going to affect it worse if you don't try something."	INNOVATOR: “I love [LAI-ART], the idea of the less you have to remember what you have - because no one likes knowing they have HIV. So, anything that allows me to think less about that during my day would be perfect for me."
Case Study 6<br><br>28YO Black Heterosexual Transwoman ATL<br><br>DELIBERATOR	HESITANT-DELIBERATOR: "It's something to think about. It's not nothing that I would, like, rush into because I honestly don't like needles."<br><br>OTHER INJECTIONS: “Now as far as the estrogen, I take that for hormone therapy (self-injection) – I’m in transition.”	ELIGIBILITY: “It's not really hard to get undetectable. When I first started Triumeq, by that third week I was undetectable. So – it's not, like, that hard."<br><br>LEAD-IN: “I don't want to hurry and switch if that's possibly going to make me feel bad. Any medicine that I start, it always makes me feel sick. I don't want to go through that again.”	INJECTION EXPERIENCED: “Because it's in your butt, it must be pretty painful.”<br><br>SECURING PRESCRIPTION: "I would think it wouldn't be just on me to change my medicine. I think it would be on my doctor to change it…If she feels I need to change it, I think it would be for the better."	MISTRUST: “It's a lot of stuff on the news, like, medicines occasionally don't agree with people. I don't want [more] health issues.”<br><br>MISTRUST: “I wish I didn't have the hassles of ADAP and Ryan White, as soon as I do it, the fastest it's turned off…I have to keep bringing all this paper and it's just frustrating.”	HIV STIGMA: "I don't know who to trust, but I just really just have extreme trust issues when it comes to knowing me personally and being all up in my business."<br><br>RESILIENCE NARRATIVE: "Yeah, that sucks [learning you have HIV], but I'm alive and I'm well."	DELIBERATOR: "I'm not saying no and I'm not saying yes to it, but it's just something that I have to think about over time."<br><br>DELIBERATOR: "I just have to maybe see from others who have been on it first."

**Black/AA**						
Case Study 7<br><br>63YO Black Heterosexual Male ATL<br><br>PRAGMATIC TO DELIBERATOR	ENTHUSIASTIC-PRAGMATIC: "Actually, I'm curious and I'm open to it. You know because it sounds good... And then not to say it's, I'm just hoping for it. You know sounds good… You know with the injection - it makes us closer to a cure."	LEAD-IN: "I'd definitely jump straight into the injection. I'll talk it over first with [my doctor]… But I've been undetectable over five years, just go straight to injection."<br><br>BRIDGE PILLS: "That does sound pretty cool, it's like, a just in case sort of thing, you know, something to fall back on. And it's not like you're going intentionally miss the appointment. But just in case."	INTEGRATION CONCERN: "More curious about forgetting because I have a very active life, you know, I'm always busy doing something as far as educating, different research, and volunteering."<br><br>SELF-MANAGEMENT: "[Controlling HIV], is something like doing exercise, you know, be consistent about it…that's why I take stuff so personally now…I do believe [using LAI-ART] would probably be okay."	TRUST IN CLINIC: "I come here to pick up my medication every 30 days. And then I come and see my doctor. And, I got cured of hepatitis C here three years ago. Yeah, like a one-stop shop for me."<br><br>TRUST IN CLINIC: "Overall, very beneficial and supportive. Like for getting more educated about HIV and being able to educate my family. And now I go to this group. Just coming here has been very beneficial mentally and health-wise."	HIV STIGMA: "I was in denial the first five years… then my father was diagnosed with AIDS. That's when I started treatment and then my father actually passed from full-blown AIDS... I didn't think that I was going to see fifty."<br><br>PRIVACY: "I used to not be, felt like I'm being ostracized or judged...[Now] if one of these females accepts me or not is on them. I just have to be honest. Took a while to get here, but I'm grateful that I did it."	DELIBERATOR: "I just have a host of questions about this. Especially something new for me. But it sounds, I mean like something worth looking into though. You know really, I'm very interested."
Case Study 8<br><br>39YO Black Gay Male CHI<br><br>PRAGMATIC TO SKEPTIC	ENTHUSIASTIC-PRAGMATIC: "It sounds like a great idea. That would be a lovely idea. You're basically saying you just inject yourself once a month. Compared to every day or, you know, forgetting to take it or anything like that…Like you can do it on the first and just keep on going about the rest of it."	LEAD-IN: "So basically gets you acclimated...and to see if it's any good for you at all....If I had any symptoms, I could just address them. I would feel pretty comfortable."<br><br>BRIDGE PILLS: "That's one of the reasons why [it would] be great to administer it yourself. You don't have interruptions or, you know, trying to find the time or get the appointment."	INTEGRATION CONCERN: "Who has the time to run to the doctor every four weeks? That would be the problem."<br><br>SELF-MANAGEMENT: "I don't know if I feel tired because of my kidney disease, or [HIV] or because of life…I can tell if something's really, really different. But you know something bad to me feels like every day [laughs]."	MISTRUST: "I don't know how they're going to do that. Some doctors don't have availability for months. That's going to be a really difficult. Unless they do it in a lab, but that would [be a] big waste. Not everyone's going to be doing it. You'd have to hire people to run the lab. It sounds like it's making it more difficult than it needs to be."	PRIVACY: "Oh, yeah. I'm open. I don't care. It's like having diabetes or something to me. [laughter]."	SKEPTICAL: "I would say, yeah, as long as I can do it myself. [laughs]."
**Latino**						
Case Study 9<br><br>64YO Latino Heterosexual Male SF<br><br>INNOVATOR	ENTHUSIASTIC-INNOVATOR: "It's very good…I would like to help the scientists to help test it, to combat this disease because I have a lot of diseases. If I had one more – that wouldn't be a problem."	LEAD-IN: "I don't have an opinion...It depends on what the doctor says...I usually don't have any problems with meds."<br><br>MONTHLY VISITS: "Honestly, I don't have any experience with that. I don't know anything. Nothing. I would like my doctor to be the one to decide…I'll do whatever my doctor tells me."	SECURING PRESCRIPTION: "It would have to be between the scientist, the one with the medication, my doctor, and myself. The doctor knows what they want to know…Whether it's right for me or not, I trust my doctor if she says, 'You're a candidate for this', I don't have a problem with that."	TRUST IN SCIENCE: "It's not going to remain stagnant. The scientists, the pharmacies always have to be working but they always have to look for candidates from our population to put their inventions into practice…But I want to be combined with my doctor because I'm a little crazy."	PRIVACY: "In my religion if you have these kinds of problems, it's because you've done something wrong. I couldn't tell anyone about it."<br><br>HIV STIGMA: "When I miss an appointment, I get really sad. I feel like an irresponsible good for nothing because I didn't know how to get myself going."	INNOVATOR: "We have to fight. If we don't, we're not going to find out. Not all types of support are equal…I am able to help a scientist find out if this medication is going to work in my body. There are different ways, and you have to fight. Just as they are fighting, I have to help out a little bit."

Case Study 10<br><br>35YO Latino Queer Male CHI<br><br>PRAGMATIC TO SKEPTICAL	ENTHUSIASTIC-PRAGMATIC: "I mean anything that gives me more autonomy in getting rid of the constant daily reminder of HIV, is great for me."	MONTHLY VISITS: "I've been getting medication for three months sometimes now. So, that possibly will not be on the horizon then…Ideally, I would go the least times possible."<br><br>BRIDGE PILLS: "I will also have to travel with pills? It's not a huge impact, but it's one issue the injection is not solving of my specific situation."	EXTENDED TRAVEL: "Still today, the fact that I have to go in every month is a pain because I spend part of the year abroad. So, how do I get the medication for all that period of time? It's always stressful."<br><br>SECURING PRESCRIPTION: "Now, I feel very well-informed. [My doctor] tells me all the options, shares her opinion, but I always feel that I am making the decision."	MISTRUST: "When I was first diagnosed, I was told that I didn't have options. And now, I think that was not true. I think I should have started my medication after my trip, and that was never an option that was offered to me. I felt that they applied a protocol to me without taking into account my actual situation. And that harmed me a lot."	PRIVACY: "Both, my colleagues, and my friends, know I'm HIV positive. They know that I take this medication, and they will know that I go to the hospital to get this. So, I have their support."	SKEPTICAL: "I will consider it... but the thing I value most at this time is flexibility with my lifestyle. If the medication is keeping me like tied down, I will not take it. So, yeah, that's a deal-breaker for me."
**Less-Engaged**						
Case Study 11<br><br>33YO Black Bisexual Male CHI<br><br>PRAGMATIC	ENTHUSIASTIC-PRAGMATIC: " I was like, "Yeah. That's good." Because, with me, I guess it will work. It would depend on the lifestyle that you have…I think it would be a lifesaver, especially for those people who have a hardship remembering to take their medicine or just too embarrassed to take it or just don't—like, who have a hard time swallowing pill."	INJECTION SITE: "It's good, but the whole injection in the booty part, that part is, ooh, I don't know…especially with us being gay…Is it going to cause a mark? That's kind of what I was worried about."<br><br>VISIT SCHEDULE: "Only thing that I would see is it would probably be timing. Luckily, my job allows me true flexibility, but I am required to travel two weeks out of the month."	SELF-MANAGEMENT: "You can get into the habit of just feeling so good and just not taking it…I had a friend. He'd been missing, like, three months. And I'm like, "What's going on?" He was like, "Yeah. I just forgot. I was feeling so good and just in the habit of just not taking it… I don't want to be in that area where I'm just so—the medicine's working and life is good, and then I have no time to maintain this."	TRUST IN PROVIDER: "I have [my doctor's] number. You know, he's very open and available. It just seems phenomenal. I recommend all my friends to come here. I really like him...And like, you know, one thing for me he was very thorough. And then he was also French, and I like the French culture…so it just made sense to, like, "Oh, I'm going with him." It just worked."	PRIVACY: "I'm just trying to focus on privacy. Those who know, know. But, again, I don't want to have nobody going—or, like, flag like, "Hey, he got it." So that's kind of why."	PRAGMATIC: "The efficiency on how it's working will supersede all, you know, if I really want to go through that. If I'm undetected and it was to keep me undetectable—you know what I'm saying—then I don't think I would worry about it."
Case Study 12<br><br>31 Black Gay Male ATL<br><br>PRAGMATIC TO DELIBERATOR	ENTHUSIASTIC-PRAGMATIC: "That would be awesome. Like just a shot once a month and that's it. I wouldn't have to worry about missing doses, or having side effects, or going through any chaos that I've been going through."	INJECTION PAIN: "If you would just get a shot once a month -it might be a little painful for an hour or two - but then you're done…I wouldn't have to worry about missing doses."<br><br>BRIDGE PILLS: "I think that sounds good because it's not penalizing whoever is taking the medicine. It's just a boost until you can get your next injection next month."	PHYSICAL VULNERABILITY: "I was off meds for like five years. My T-cell count was like a one. This injection wouldn't have helped me back then. But with time and taking my medicine, I think I'd be a good candidate. But right now, no,"<br><br>COMPETING PRIORITY: "If they schedule me on a day that I have work it might be a little challenging. I might have to reschedule."	TRUST IN PROVIDER: "She's kind of like a big sister. She's not really my sister, but I just have this feeling that I can talk to her about anything if I have a problem or an issue. And not just with my medication or my health. If I have something that I need to discuss with her, she has opened that door so that I can come in and talk to her whenever."	HIV STIGMA: "I'm just being hard-headed. My pills are like tic-tacs, I should have no problem taking them, but I just don't …I have something in my head that tells me that when I'm feeling well, I don't need them anymore, so I stop taking them...No valid reason…I've been trying to work with the therapist to try to get some things figured out."	DELIBERATOR: "For now, until I get to undetectable for a long period of time, then I would stick with the oral. But once things progress, I get above 200, and things are going well, then I am not opposed to the injections. I would be glad."
